# Huge epithelial nonparasitic splenic cyst: A case report and a review of treatment methods

**Published:** 2016

**Authors:** Bahman Farhangi, Arezo Farhangi, Alireza Firouzjahi, Babak Jahed

**Affiliations:** 1 Department of Surgery, Babol University of Medical Sciences, Babol, Iran.; 2 Semnan University of Medical Sciences, Semnan, Iran.; 3 Department of Pathology, Babol University of Medical Sciences, Babol, Iran.; 4 Ayatollah Rohani Hospital, Babol University of Medical Sciences, Babol, Iran.

**Keywords:** Cysts diagnosis, Cysts surgery, Splenic diseases diagnosis, Splenic diseases surgery.

## Abstract

**Background:**

Splenic cysts are rare in all age groups and there are a few reports in the world literature. Primary cysts occur most frequently in children and young adults, comprising around 25% of all nonparasitic splenic cysts. Various techniques are suggested for the treatment of splenic cysts. In this case report, a huge epithelial splenic cyst in a 17-year-old female is presented and different treatment methods of splenic cysts are evaluated.

**Case Presentation:**

A 17-year-old female presented with progressive abdominal mass in left upper quadrant associated with abdominal pain and food intolerance of duration of several months. There was no history of trauma. On physical examination, there was a huge mass located in the upper left side of abdomen. Computerized tomography scan revealed that a large cystic lesion had occupied the spleen with dimensions of 32x21xI5.6 cm. After patient preparation laparotomy was performed and complete cyst excision was done with splenectomy, patient was discharged after 2 days. This is a report of a case of epithelial splenic cyst of the spleen in a 17-year old female.

**Conclusion:**

The management of splenic cysts continues to evolve and the optimum treatment of patients with nonparasitic splenic cysts is controversial, as a principle preservation technique of the spleen with minimally invasive methods such as laparoscopy is preferred to splenectomy with the exception of very large cysts and when splenic hilum is involved in cyst wall. However, significant cyst recurrences were encountered with these techniques. Recently open partial splenectomy has been proposed as a safe and effective method in the management of NPSCs it ensures complete cyst removal, lack of cyst recurrence, and preservation of the spleen functions.

**S**plenic cysts ICD-I0 code D 734 are rare lesions in all age groups and there are limited reports in the world literature. Nonparasitic splenic cysts (NPSCs) are uncommon and since 1993 approximately 800 cases have been reported since 1993(1). Splenic cysts are classified as 2 main categories. 1- Parasitic disease particularly echinococcosis which is the most common etiology for splenic cysts worldwide (2). 2- Nonparasitic which consists of primary and secondary cysts. Primary or true cysts have epithelial lining (epidermoid, dermoid and mesothelial) or endothelial cover (hemangioma, lymphangioma) (3). Primary or congenital cysts are encountered more commonly in children and young adults comprising 25% of all nonparasitic cysts (4).

These cysts are mostly asymptomatic and are usually an incidental finding during abdominal ultrasonic examination (5). Secondary splenic cysts result from trauma, in which they lack epithelium and are called pseudocysts (2). Clinical presentation of splenic cysts is more common with abdominal pain mostly in the left upper quadrant associated with nausea and vomiting due to gastric pressure and also splenomegaly or mass in the upper abdomen. Large splenic cysts are susceptible to complications such a hemorrhage, rupture after abdominal trauma or infection (6). In this report a case of congenital epithelial splenic cyst in a 17-year-old female who was referred with abdominal pain and abdominal mass is presented. 

## Case presentation

A 17-year-old female patient presented in March 2009 due to progressive enlargment of a mass in the upper abdomen with pain in this particular area and food intolerance in the last few days prior to admission. The patient did not have any history of abdominal trauma. On physical examination, a large mass was detected in the left upper part of abdomen. 

**Figure 1:**
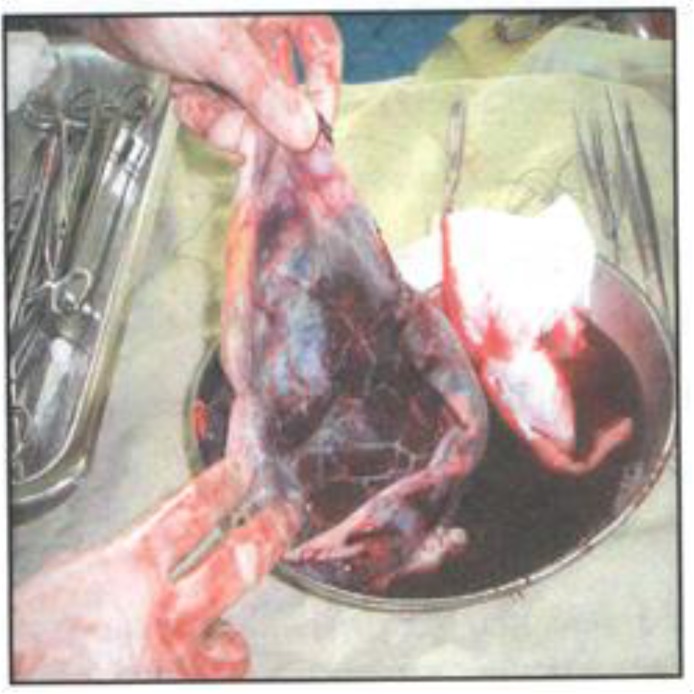
Huge epithelial splenic cyst

Ultrasonography and computerized tumography examinations showed the spleen to have a large cystic lesion of 32 x 21 x 15.6 cm, while the liver and kidneys were normal. After admission, lab tests to rule out hydatid cyst were performed that were negative. After preventive vaccination, laparatomy was done; operative finding was a very large splenic cyst containing more than 1 liter of serous fluid (fig 1). 

Since there was little healthy splenic parenchyma left and splenic hilar vessels were involved in the cyst wall and it was not possible to preserve the spleen, thus complete cyst excision with splenectomy was done. Specimen was sent to a pathology laboratory. The patient was discharged after 3 days in good condition. 

**Figure 2:**
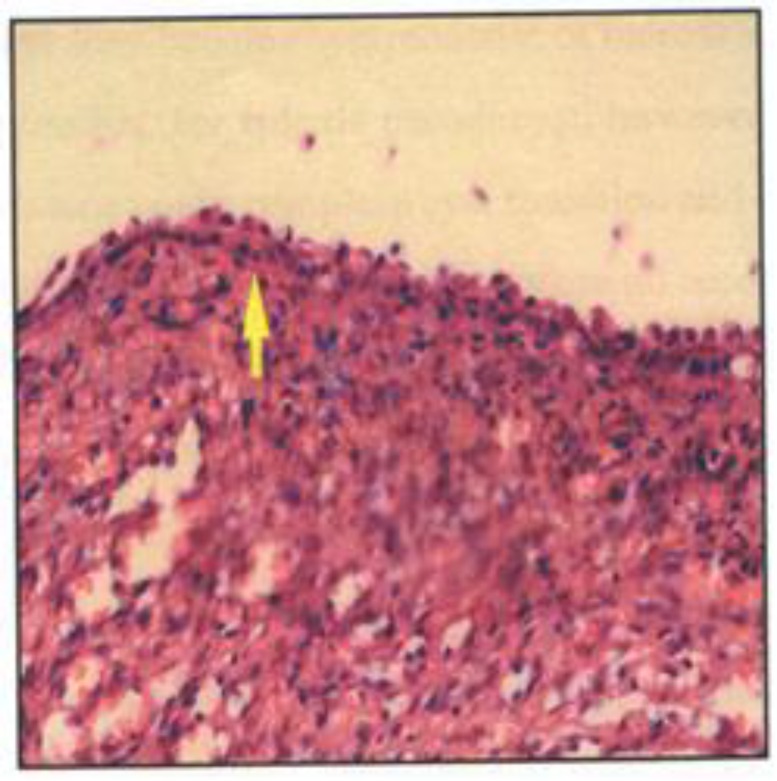
The cyst wall is surfaced by layers of cuboidal (mesothelial-like) epithelium

**Pathological report**: Specimen includes cystic spherical spleen weighing 320 gram. Cyst wall is thick and gray colored, which internal surface is irregular with fibrotic septums. Spleen parenchyma with a large cystic lesion was observed. Internal lining of cyst wall was associated with trabeculation. Parts of internal surface is covered by two to several layers of cuboidal mesothelial like epithelium, however in most of the areas, cyst wall is completely hyalinized and thickened without cellular lining (fig 2). Splenic parenchyma is associated with red pulp congestion and white pulp atrophy. Pathologic diagnosis: splenic epithelial cyst. 

## Discussion

Management of nonparasitic splenic cysts **(NPSCs) **continues to evolve and optimal treatment of these patients is controversial (1, 5, 7). The standard treatment of splenic cysts was splenectomy in the past, but concerns on the possibility of post splenectomy infection caused most surgeons to adopt techniques to save as much splenic tissue especially in pediatric age group (1, 7). Treatment of NPSCs depends on the presence or absence of clinical findings, asymptomatic cysts can be followed by ultrasonography to exclude significant expansion up to 10 cm diameter. Patients with large cysts should be advised of the risk of cyst rupture with even minor abdominal trauma if nonoperative management is adopted. Laparoscopic excision of (NPSCs) is associated with high recurrence rates in children. In one study by Fisher, cyst recurrence occurred in 7 patients out of 8 at a median of 9.4 months after initial surgery (8). Decapsulation, (near total excision of cyst wall) is one of the other methods of treatment of splenic cysts, Mertens reported cyst recurrence in 4 out of 7 patients after cyst decapsulation of primany splenic cyst (9). It is recommended that in case of partial decapsulation, the omentum is then packed into the remaining shell of the cyst. 

Percutaneus drainage with injection of a sclerosing agent has complications and significant recurrence rate because a portion cyst wall internal lining has been left intact (7). Majority of (NPSCs) are caused by blunt abdominal trauma following the adoption of nonoperative management of blunt splenic injury in stable patients in the last few decades, the incidence of pseudocysts of spleen has increased (1). Approximately 75% of the (NPSCs) are of the false cysts (secondary) type with no epithelial lining (4). 

Intraparenchymal hemorrhage of spleen results in hematoma formation, after some time, a fibrous capsule is formed and with pigment reabsorbtion leads to a serous cyst. Splenic pseudocysts are asymptomatic in 30 to 60% of cases and with passage of time some of these cysts may resolve spontaneously suggesting they may be safely followed serially by ultrasonography. Surgical treatment is indicated in case they become symptomatic or have increased in size. 

It is general consensus to avoid splenectomy if possible, for splenic pseudocyst, however it is not clear which spleen sparing procedure is the best treatment; only complete cyst resection and a portion of contiguous splenic parenchyma assures that no cyst remnant remains and therefore, no recurrence of the splenic pseudocyst will occur. (10). Treatment of small symptomatic (NPSCs) is recommended by cyst excision with splenic preservation and large cysts can be unroofed; both operations can be performed laparoscopically, however in one study by Schier et al in 2007, laparoscopic unroofing of splenic cysts were associated with a high rate of cyst recurrences in 9 patients out of 14 treated with this technique, the cysts recurred at intervals ranging from 6 to 12 months. 

This study recommends to prevent cyst recurrence by peeling off of internal cyst wall layer or partial splenectomy with complete cyst excision, (11) nevertheless many surgeons do not have adequate experience with partial splenectomy and control of bleeding from incised surface of spleen can be difficult. Splenectomy can be considered as a reasonable option in large cysts when small splenic parenchyma has remained or splenic hilar vessels are involved in cyst wall making splenic preservation difficult (1, 12, 13). 

Machenzie et al, reported laparoscopic decapsulation of congenital splenic cyst using harmonic scalpel, diathermy, endoshears in 3 patients and recommended this technique as an effective and safe method, of course a larger patient group is needed for evaluation of incidence of cyst recurrence (13). Szczepanik et al conducted open partial splenectomy in 10 patients with (NPSCs). Spleen parenchyma was cut with the aid of a LigaSure instrument and bleeding from the transected splenic parenchyma was secured with argon plasma coagulation and absorbable tape sutures or oxidized cellulose. The postoperative course was uneventful in all cases. He proposed open partial splenectomy as a safe and effective method in the management of (NPSCs) it ensures complete cyst removal, lack of cyst recurrence, and preservation of the spleen functions (10). 

## Conclusion

The management of splenic cysts continues to evolve and the optimum treatment of patients with nonparasitic splenic cysts is controversial, As a principle, splenic preservation techniques with minimal invasive methods such as laparoscoscopy is prefered to splenectomy with the exception of very large cysts and when splenic hilum is inovled in cyst wall. Nonetheless, significant cyst recurrences were encountered with these techniques. Recently open partial splenectomy has been proposed as a safe and effective method in the management of (NPSCs) ensuring complete excision the lack of cyst recurrence, and preservations of splenic functions. 
